# Butyrate mitigates metabolic dysfunctions via the ERα-AMPK pathway in muscle in OVX mice with diet-induced obesity

**DOI:** 10.1186/s12964-023-01119-y

**Published:** 2023-05-04

**Authors:** Qingsong Fu, Tiantian Li, Chen Zhang, Xiaotian Ma, Liying Meng, Limin Liu, Kai Shao, Guanzhao Wu, Xing Zhu, Xiaoyun Zhao

**Affiliations:** 1 Department of Medical Experiment Center, Qilu Hospital (Qingdao), Cheeloo College of Medicine, Shandong University, 758 Hefei Road, Qingdao, 266035 Shandong China; 2 Department of Qingdao Key Lab of Mitochondrial Medicine, Qilu Hospital (Qingdao), Cheeloo College of Medicine, Shandong University, Qingdao, 266035 Shandong China; 3 Institute of Biomedical Engineering, West China School of Basic Medical Sciences & Forensic Medicine, Sichuan University, Chengdu, 610041 China; 4 Department of Pathology, Qilu Hospital (Qingdao), Cheeloo College of Medicine, Shandong University, Qingdao, 266035 Shandong China

**Keywords:** Metabolic syndrome, Sodium butyrate, Oestrogen receptor alpha, Energy metabolism, Lipid metabolism, Mitochondrial function

## Abstract

The higher prevalence of metabolic syndrome (MetS) in women after menopause is associated with a decrease in circulating 17β-oestradiol. To explore novel treatments for MetS in women with oestrogen deficiency, we studied the effect of exogenous butyrate on diet-induced obesity and metabolic dysfunctions using ovariectomized (OVX) mice as a menopause model. Oral administration of sodium butyrate (NaB) reduced the body fat content and blood lipids, increased whole-body energy expenditure, and improved insulin sensitivity. Additionally, NaB induced oestrogen receptor alpha (ERα) expression, activated the phosphorylation of AMPK and PGC1α, and improved mitochondrial aerobic respiration in cultured skeletal muscle cells. In conclusion, oral NaB improves metabolic parameters in OVX mice with diet-induced obesity. Oral supplementation with NaB might provide a novel therapeutic approach to treating MetS in women with menopause.

**Graphical Abstract:**

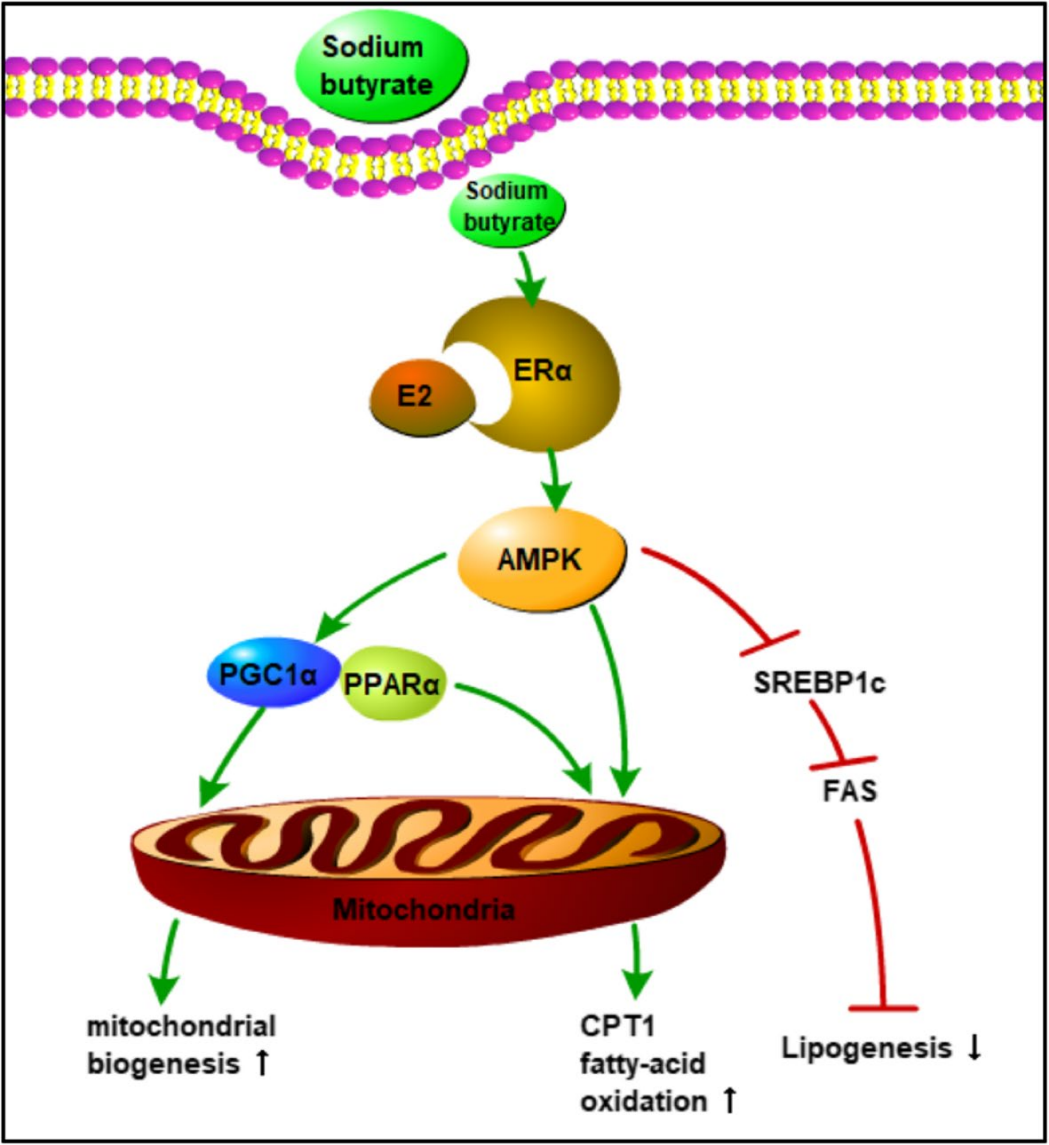

Video Abstract

**Supplementary Information:**

The online version contains supplementary material available at 10.1186/s12964-023-01119-y.

## Introduction

Menopause is often associated with a higher incidence of metabolic syndrome (MetS) and related comorbidities, including obesity, insulin resistance, dyslipidaemia, and type 2 diabetes [1], which suggests that the female steroid hormone oestrogen mediates metabolic homeostasis [2]. Previous studies in high fat-fed ovariectomized mice have shown the association between oestrogen deficiency and exaggerated metabolic dysfunctions, and oestrogen treatment improved metabolic homeostasis by increasing energy expenditure and insulin sensitivity in these mice [3, 4]. Although oestrogen replacement therapy has been used in women who experience intolerable symptoms of menopause and severe disorders such as postmenopausal osteoporosis, the increased risks associated with hormonal therapy of coronary heart disease, thromboembolic disease, stroke, and certain gynaecological cancers have limited its long-term use in a larger population [5]. These side effects have made it unsuitable to control or prevent MetS in women in broad age groups. Therefore, a search for a safe, non-oestrogen clinical approach is necessary.

Butyrate, a short-chain fatty acid (SCFA) produced from anaerobic bacterial fermentation of dietary fibre in the colon, not only serves as a main energy substrate for colonic epithelial cells but also as a signal to regulate various cellular activities. Several studies have reported that butyrate alleviates obesity and insulin resistance in high-fat diet-fed (HFD) mice by increasing energy expenditure and improving the utilization of fatty acids [6–9] by activating mitochondria. As skeletal muscle comprises approximately 50% of the body weight and accounts for 80% of insulin-stimulated glucose disposal, the metabolic capacity of skeletal muscle plays a vital role in maintaining the whole-body energy balance and glucose and lipid homeostasis [10]. Recent research has suggested that the increased prevalence of obesity and MetS might be driven by the effects of physical inactivity and a sedentary lifestyle causing skeletal muscle insulin resistance and a decrease in glucose disposal, a major risk factor for developing type 2 diabetes [11, 12]. Because the skeletal muscle from female rats was found to have greater mitochondrial mass and oxidative phosphorylation capacity than that from their male counterparts [13], decreases in mitochondrial function in skeletal muscle might be the underlying cause of the rise in insulin resistance and metabolic dysfunctions in response to oestrogen deficiency; butyrate supplementation could restore metabolic homeostasis by directly improving mitochondrial functions in skeletal muscle.

Oestrogen receptors (ERs) are ligand-dependent transcription factors that form dimers to interact with classical oestrogen response elements (EREs) in the promotor regions of oestrogen-regulated genes. ERs are regulated by growth factors via intracellular kinase signalling pathways and ligand-independent pathways [14, 15]. Two main forms, ERα and ERβ, were broadly expressed in the central and peripheral tissues, including adipose, skeletal muscle, liver, and immune cells. Among them, clinical evidence and rodent experiments in males and females with ERα variants all develop features of metabolic syndrome, including obesity, glucose intolerance, and insulin resistance [16, 17]. Oestrogen receptor alpha in skeletal muscle has drawn specific attention given its effects on maintaining mitochondrial function and energy metabolism in females. A study using muscle-specific ERα knockout mouse model showed that the impairment in ERα is associated with decreases in muscle mitochondrial oxidative capacity and increases in muscle lipid accumulation [18]. Since 17β-oestradiol regulates mitochondrial function and energy metabolism mainly by activating AMP-activated protein kinase (AMPK) via ERα, while butyrate improves glucose and fat metabolism via G protein-coupled receptors and monocarboxylate transporters, acting as a histone deacetylase and by passing ERα to activate AMPK and other pathways, it is reasonable to speculate that the metabolic effects of butyrate are dependent on the activation of ERα [19–22]. Therefore, we hypothesized that dietary butyrate could restore metabolic homeostasis in mice with oestrogen deficiency by activating the AMPK signalling pathway depends upon ERα in skeletal muscle. In the current study, the effects of sodium butyrate (NaB) on preventing and restoring dietary-induced metabolic dysfunctions under oestrogen deficiency were studied using high fat-fed OVX mice. The related molecular parameters were measured using cultured muscle cells to help interpret the mechanisms of butyrate in promoting energy metabolism in skeletal muscle.

## Materials and methods

### Clinical studies

The study was approved by the ethics committee of Shandong University Qilu Hospital. Written informed consent was obtained from all participants prior to data collection. A simple inclusion and exclusion method was used to exclude malignant tumours or metabolic diseases such as thyroid diseases, pulmonary tuberculosis, or pregnancy. There were no missing records of demographic characteristics, and the MetS scores had five items. The study recruited adults aged 30 to 69 years from Qilu Hospital Medical Center (2013–2021) for physical examinations. A total of 55,887 adults, including 25,708 females and 30,179 males, were recruited for the study. All the patients were divided into four age groups: 30 to 39 years old (F 9,176 and M 11,276); 40 to 49 years old (F 6,516 and M 7,310); 50 to 59 years old (F 6,098 and M 7,199); and 60 to 69 years old (F 3,918 and M 4,394). MetS was diagnosed using the Chinese Diabetes Society diagnostic criteria when any three or more of the following risk parameters were presented [23, 24]. Blood samples obtained from 183 female patients were further extracted and measured for serum oestradiol. A test for a linear trend was performed between the decline in oestradiol and the increasing prevalence of MetS with age. Correlation analyses between MetS prevalence and changes in blood oestradiol were performed using t tests and logistic regression analysis. Since oestradiol concentrations had a positively skewed distribution, logarithm transformation was applied to normalize the data.

### Animals and treatment

All animal procedures were approved by the Institutional Animal Care and Use Committee of Shandong University Qilu Hospital. Female C57BL/6 J mice (6 weeks old) were purchased from Jinan Pengyue Laboratory Animal Breeding Co., Ltd. All the mice were housed in a controlled environment (22 ± 1 °C, 50 ± 5% relative humidity, 12/12-h light/dark cycle) and were given free access to food and water. After two weeks of acclimation to the study environment, the mice were divided into four groups (*n* = 12): 1) control group, fed with a regular diet (D12450B, Research Diets Inc, USA) (CON group); 2) sham-operated group, fed with a high-fat diet (HFD, D12492, Research Diets Inc, USA) (SH group); 3) ovariectomized group (OVX), fed with HFD (OH group); and 4) NaB-treated group, OVX and fed with HFD (OHB group). Ovariectomy and the sham operation were performed at eight weeks old. All mice were switched from a regular diet to a HFD at ten weeks old except the control group, which was continuously kept on regular chow. After four weeks on the HFD, OVX mice were divided into two groups and received a daily gavage of NaB (200 mg per kg body weight) or vehicle in saline for eight weeks. At 22 weeks of age, mice were sacrificed by cervical decapitation after 12 h of fasting. The eyeball was removed to collect trunk blood, and gonadal parametrial white adipose tissue, liver, and muscle were quickly removed, frozen in liquid N2, and stored at -80 °C until analysis was performed.

### Indirect calorimetry

Mice were singly housed in the metabolic chambers for 24 h to acclimate to the testing environment before the experiment began using an indirect calorimeter (Promethion, Sable Systems International, USA). The assessments were conducted continuously for 48 h (containing two light and two dark cycles) while mice were given free access to food and water. Oxygen consumption (VO_2_), carbon dioxide production (VCO_2_), respiratory exchange ratio (RER), food intake, and in-cage spontaneous movement were measured. Energy expenditure (EE) was calculated using the equation EE = 60*(0.003941*VO_2_ + 0.001106*VCO_2_). Differences in EE were analysed using analysis of covariance (ANCOVA) with body-weight treated as an independent variable [25].

### Cell culture

C2C12 cells (American Tissue Culture Collection; ATCC®CRL-1772) were cultured as described previously. Briefly, C2C12 cells were maintained in Dulbecco’s modified Eagle’s medium (DMEM, Thermo Fisher Scientific) supplemented with 10% foetal bovine serum, penicillin (50 units/ml), and streptomycin (50 mg/ml) to confluency (80–90%). The medium was transferred to differentiation medium (DM) with 2% horse serum, which was replenished every day. Four days after differentiation, the C2C12 cells had fused into myotubes and were treated with 1) vehicle, 2) glucosamine (GlcN, 15 mM), 3) sodium butyrate (2 μM), 4) oestrogen (10 nM) or 5) the oestrogen receptor alpha inhibitor fulvestrant (ICI 182,780) (10 μM) for a specified time.

### Histology

Liver and adipose tissues were fixed in 10% (w/v) formaldehyde and embedded in paraffin (5-μm thick). Skeletal muscle was embedded in OCT-Freeze Medium (10-μm thick). All tissue sections were stained with haematoxylin–eosin staining (HE) for morphological analysis. Liver and muscle sections were stained with Oil Red O for quantitative and qualitative measurement of fat depots. Images were captured using a microscopic imaging system (DM2500, Leica, Germany) with a magnification of 2 × 100. The steatosis area and Oil Red O-positive area were quantitated by ImageJ 1.8 s (provided in the public domain by the National Institutes of Health, Bethesda, MD, USA) (*n* = 3, per 3 fields).

### Glucose tolerance test and insulin tolerance test

A glucose tolerance test (IPGTT) was performed seven days before the mice were sacrificed (*n* = 6). After an overnight fast (14 h), mice were given an IP injection of glucose (2.5 g/kg in saline). Blood samples were collected via the tail vein, and blood glucose was measured using a glucometer (ACCU-CHECK Performa, Roche) before and after the injection at 0, 15, 30, 60, 90, and 120 min. Insulin tolerance tests (IPITTs) were performed three days before the mice were sacrificed in the above mice (*n* = 6). After a fast (4 h), mice were given an IP injection of insulin (0.75 U/kg in saline). Blood samples were collected and measured as described above. At 22 weeks, mice were sacrificed to collect blood samples for the concentrations of fasting glucose and insulin measured by using the Hitachi 7600–020 clinical analyser (Hitachi, Tokyo, Japan) or Enzyme-Linked Immunoassay (ELISA) kits (Neobioscience Technology Co., Ltd., Shenzhen, China) according to the manufacturer’s instructions.

### Quantitative real-time reverse transcription-polymerase chain reaction (RT‒PCR)

Total RNA was extracted from skeletal muscle tissues using TRIzol reagent (Life Technologies, Gibco, USA). Approximately 50 mg of the tissue was minced well and lysed with 1 mL of TRIzol reagent, followed by the addition of 20% v/v chloroform. After centrifugation, the water phase was used for purification, followed by the addition of the same volume of isopropanol. The RNA precipitate was extracted and resuspended in RNase-free water for further processing. The RNA was reverse-transcribed into cDNA using the all-in-one TM First-Strand cDNA Synthesis Kit (Gene Copoeia, Rockville, USA). Gene expression was measured using SYBR Green in Real-time PCR (Bio-Rad, Hercules, CA, USA). Data were calculated by the 2-∆∆Ct quantification method after normalization to GAPDH. Cytochrome c oxidase subunit 1 (Cox1) was used as a marker for mtDNA, and cyclophilin A was used as a marker for nuclear DNA. The -∆∆Ct (Cox1 to cyclophilin A) represented the relative mtDNA content. The primers are listed in the Supplementary Material (Table 1).

### Western blot analysis

Total cell lysates were prepared from either muscle tissue or cultured muscle cells using lysis buffer. Protein concentrations were determined by the bicinchoninic acid (BCA) assay (Pierce Biotechnology, Rockford, IL). Equal amounts of protein were separated by SDS‒PAGE and transferred to PVDF membranes. The following antibodies were used to detect the protein of interest: PPARα (Proteintech), PGC1α (Proteintech), AMPKα/p-Thr172 (Cell Signalling), ERα (Proteintech), IRS1/p-Ser307 (Cell Signalling), AKT/p-Thr308 (Cell Signalling), and GAPDH (Abcam). The band densities were quantified using ImageJ 1.8 s.

### Cellular metabolic rate

Analysis of energy metabolism in intact C2C12 cells was performed using the Seahorse XFe24 Extracellular Flux Analyzer (Seahorse Bioscience/Agilent, Santa Clara, CA). Seahorse microplates were seeded at 50,000 cells per well. After differentiating for four days, cells were further induced by the addition of glucosamine without serum and were incubated for 24 h, followed by the addition of NaB or vehicle and further incubation for 24 h. Before the experiment, the cells were washed, and 500 μL of medium (102,353–100, Seahorse XF Base Medium) was added containing 2 mM L-glutamate, 1 mM pyruvate, and 10 mM glucose and equilibrated at 37 °C in a CO2-free incubator for 45 min to 1 h. Cellular oxygen consumption rates (OCRs) were measured during the experimental course beginning at baseline, followed by serial injections of the F0 F1-ATPase inhibitor oligomycin (1.5 μM), mitochondrial uncoupler FCCP (2 μM), and a combination of the complex I and III inhibitors rotenone and antimycin A (total 0.5 μM). The coupling efficiency was calculated as the fraction of basal mitochondrial OCR used for ATP synthesis (ATP-linked OCR/basal OCR). Data were normalized as the oxygen consumption per minute relative to the protein content of the incubation. The extracellular acidification rate (ECAR) was measured with glutamine (2 mM) supplemented in the assay medium. The measurements were performed at baseline and after injection of glucose (10 mM), oligomycin (1 μM), and 2-DG (50 mM).

### Confocal microscopy

C2C12 cells were seeded into µ-Slide eight wells (Ibidi, Germany). GLcN and NaB were treated as previously described. After being washed three times with PBS, the seeded cells were fixed with 10% (w/v) formaldehyde for 15 min. Cells were then washed three times with PBS and permeabilized with 0.1% (v/v) Triton X-100 in PBS for 15 min. Cells were blocked in 5% (w/v) bovine serum albumin buffer for 60 min and incubated with ERα antibody (1:50 diluted in PBS, Santa Cruz) and AMPK antibody (1:200 diluted in PBS, Abcam) overnight at 4 °C. After incubation, the cells were washed three times with PBS, incubated with fluorophore-conjugated secondary antibodies (goat anti-mouse, Abcam, ab150119 Alexa Fluor 647; goat anti-rabbit, Abcam, ab150081 Alexa Fluor 488) for 1 h at room temperature and washed three times with PBS. Finally, the cells were incubated with DAPI (Solarbio) for 15 min at room temperature and subjected to image analysis using a confocal laser scanning microscope (Leica). The relative intensity of fluorescence (mean) in the indicated groups was quantified using ImageJ 1.8 s.

### Statistical analysis

All data are presented as the mean ± standard error of the mean (SEM). Differences between two groups were analysed using the two-tailed unpaired t test, and multiple group comparisons were analysed by one-way ANOVA followed by Tukey’s post hoc test. Differences at *p < 0.05, **p < 0.01, and ***p < 0.001 were considered statistically significant. All statistical analyses were performed using GraphPad Prism 8.0 software (La Jolla, CA).

## Result

### Age- and sex-specific characteristics related to the prevalence and risk indicators of metabolic syndrome

A total of 25,708 females and 30,179 males were recruited to compare the differences in the prevalence and risk indicators of metabolic syndrome in four age groups. The results showed that the prevalence of metabolic syndrome (MetS) is sex-specific with age. The prevalence in the two sex groups increased with age: males (10.32% to 36.25%) and females (1.18% to 21.23%). Female groups showed much lower prevalence in the 30 to 39 and 40 to 49 age groups than males (1.18% vs. 10.32%; 3.38% vs. 21.39%), while the prevalence in the female 50 to 59 age group and 60 to 69 age group increased rapidly and closely approximated those of the male groups (11.24% vs. 31.51%; 21.23% vs. 36.25%). Notably, little fluctuation in the incidence was observed in males older than 50 years, while a twofold increase was observed in females (Figure S1A). Next, the parameters of risk factors related to MetS were counted in the male and female age groups (Figure S1B-H). All parameters of the MetS group were significantly higher than those of the normal control group (data not shown). Statistical differences were observed in body mass index (BMI), total cholesterol (TC), low-density lipoprotein (LDL), and blood glucose among individuals of both sexes with MetS after 50 years of age, as well as high-density lipoprotein (HDL) and triglycerides (TG) in all age groups. There was no significant difference in glycated haemoglobin A1c. All the results suggest that a sex-related specific factor accompanied by ageing may be critical for metabolic syndrome. Serum oestradiol was further measured in 183 female patients. The concentrations of serum oestradiol showed a downward trend with age in females (Figure S1I). Furthermore, the correlation analysis results showed that MetS was significantly associated with serum oestradiol levels in females (*P* < 0.05) (Figure S1J). In univariate logistic regression analysis, the concentration of oestradiol was a protective factor against MetS (OR = 0.535, 95% CI: 0.314–0.912, *P* = 0.0216).

### Sodium butyrate reduces body weight and improves insulin sensitivity

**Fig. 1:**
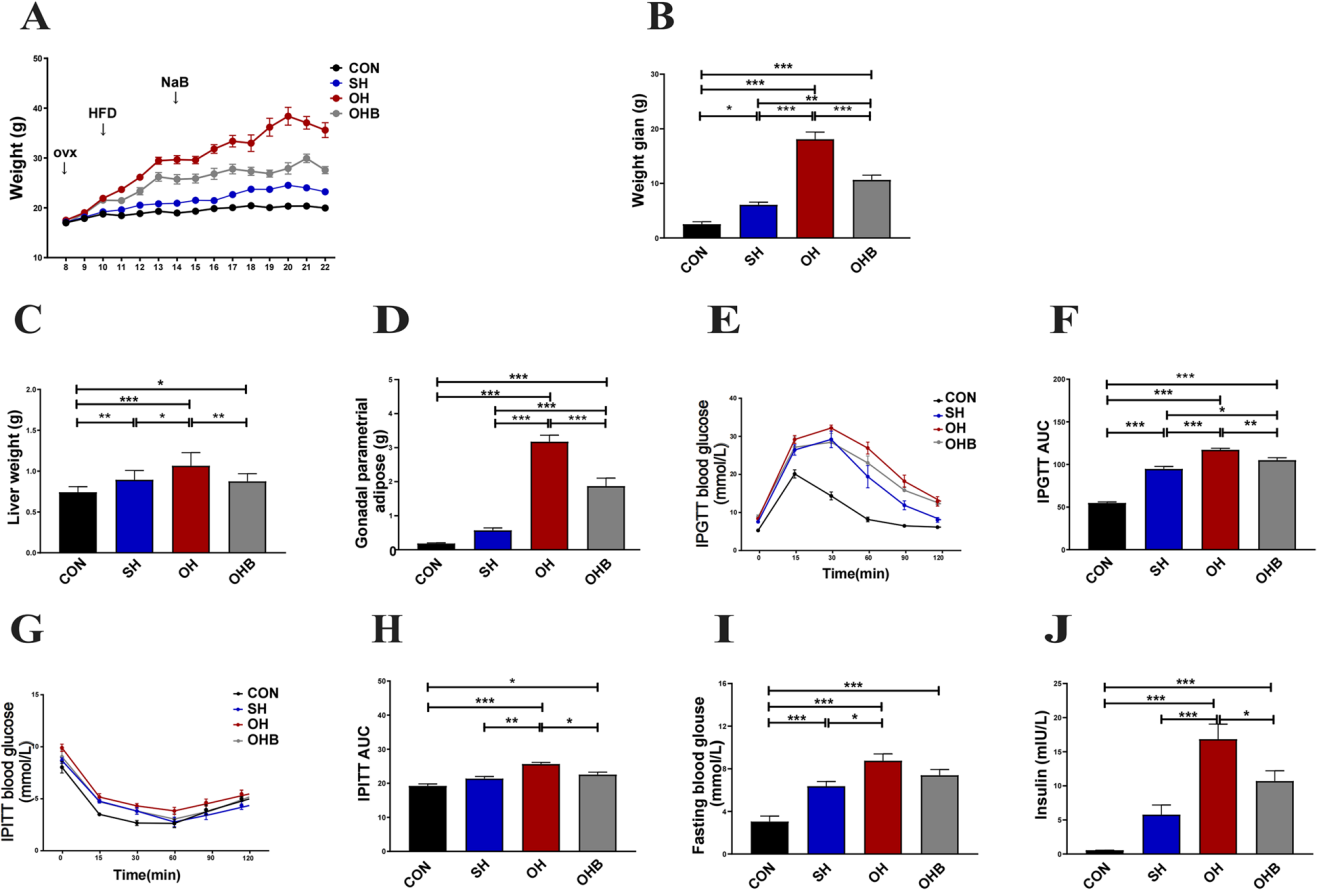
Sodium butyrate reduces body weight and improves insulin sensitivity. **A** Body weight curve (*n* = 10–12). **B** Weight gain (*n* = 10–12). **C** Liver weight (*n* = 10–12). **D **Gonadal parametrial adipose tissue weight (*n* = 10–12). **E** Mean blood glucose levels following IPGTT (*n* = 6). **F** Area under the glucose-time curve of the IPGTT (AUC) (*n* = 6). **G** Mean blood glucose levels following the IPITT (*n* = 6). **H** Area under the glucose-time curve of the IPITT (AUC) (*n* = 6). **I** Fasting serum blood glucose concentrations (*n* = 9–11). **J** Fasting serum insulin concentrations (*n* = 9–11). CON: normal control diet, SH: sham operated + HFD, OH: OVX + HFD, OHB: OVX + HFD + NaB. Data are the means ± SEMs, and the data were analysed by ANOVA (*p* < 0.05), *p < 0.05, **p < 0.01, ***p < 0.001

Next, ovariectomized mice were used to simulate female menopause in which the circulating concentration of oestradiol was absolutely reduced. The body weight, liver mass, and fat mass in OH mice were significantly higher than those in SH mice. A protective effect on these parameters was confirmed by NaB treatment (Fig. 1A-D). Furthermore, we found that the blood glucose curves of OH mice were significantly increased after intraperitoneal glucose injection compared with SH mice, indicating that OVX induced more severe impaired insulin sensitivity, which was reversed by NaB treatment (Fig. 1E-F). Consistent with the above findings, NaB treatment significantly enhanced the intake and utilization of glucose with insulin stimulation or fasting (Fig. 1G-J). These results indicated that NaB improved obesity and insulin sensitivity after oestrogen deficiency.

### Sodium butyrate increases energy expenditure in high-fat diet-fed ovariectomized mice

**Fig. 2:**
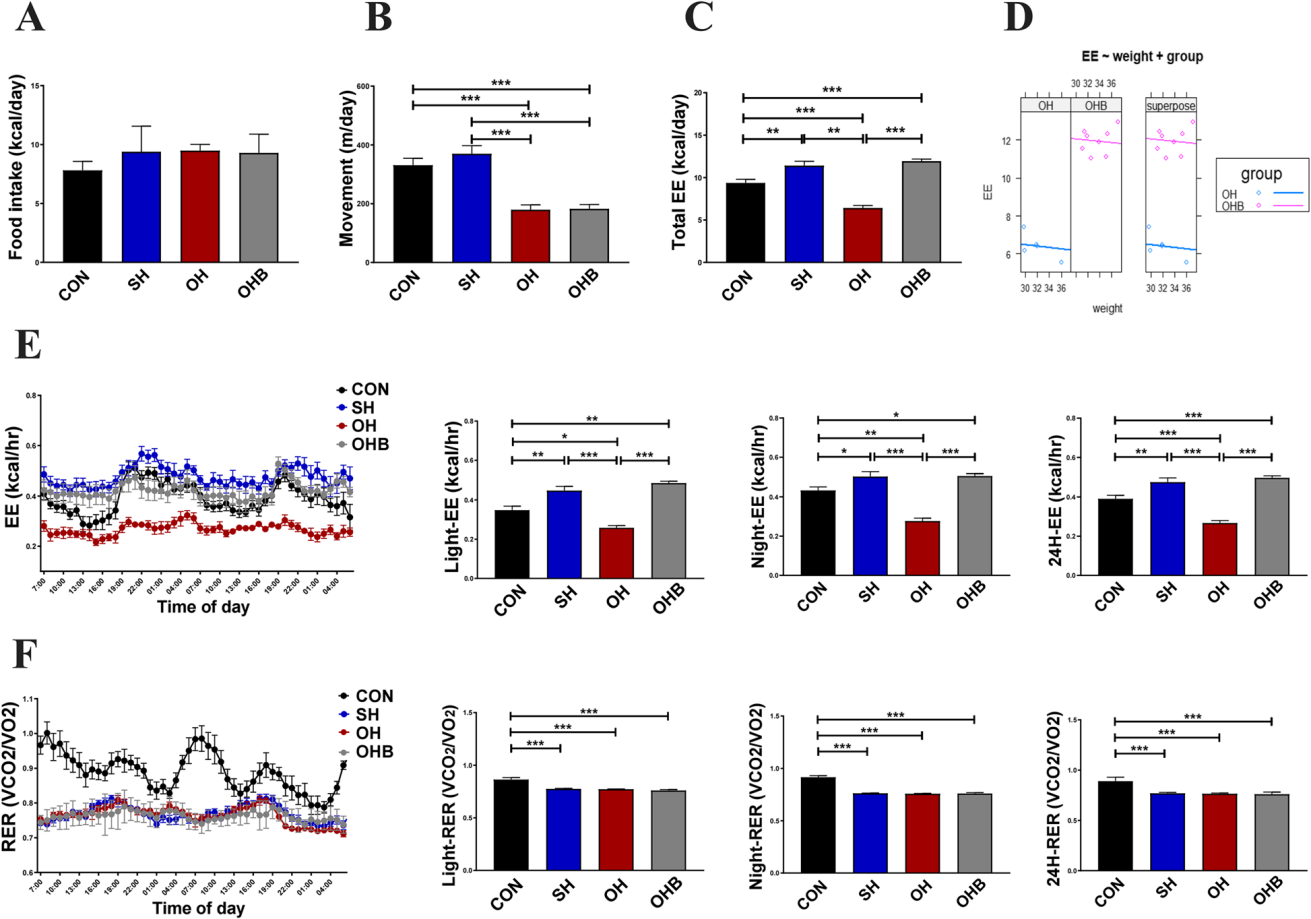
Energy metabolism in response to sodium butyrate. **A** Food intake, (**B**) movement, (**C**) total energy expenditure (EE), and (**D**) corrected energy expenditure. **E–F, **(**E**) Rate of energy expenditure (EE) and (**F**) the respiratory exchange ratio (RER). The data were calculated as the average of two days for the light and dark cycles or for the total two days (*n* = 5–8). CON: normal control diet, SH: sham operated + HFD, OH: OVX + HFD, OHB: OVX + HFD + NaB. Data are the means ± SEMs, and the data were analysed by ANOVA (*p* < 0.05), *p < 0.05, **p < 0.01, ***p < 0.001

The in vivo energy balance of the mice, i.e., food intake, in-cage activity, oxygen consumption, CO_2_ production, and the respiratory exchange ratio, were studied using an indirect calorimetry system (Sable). After preacclimation for 24 h in the metabolic chambers, the metabolic parameters of the mice were continuously measured for 48 h. Total caloric intake was not different among the four groups (Fig. 2A), while the in-cage activity was significantly reduced in the OH and OHB groups compared to the control and sham groups, with no difference between the OH group and the OHB group (Fig. 2B), suggesting that energy expenditure induced by activity was decreased in oestrogen-deficient mice and that NaB treatment had no effect on activity. Total energy expenditure was significantly decreased in the OH group compared with the SH group, but it was rescued in mice treated with NaB (OHB group) (Fig. 2C-E). Finally, the respiratory exchange ratio was only different between the mice fed different diets. The same respiratory ratio among the HFD groups suggested that both ovariectomy and butyrate treatment caused minor changes in substrate preference between fat and carbohydrate metabolism (Fig. 2F).

### Sodium butyrate reduces lipid deposition in serum and tissue

**Fig. 3:**
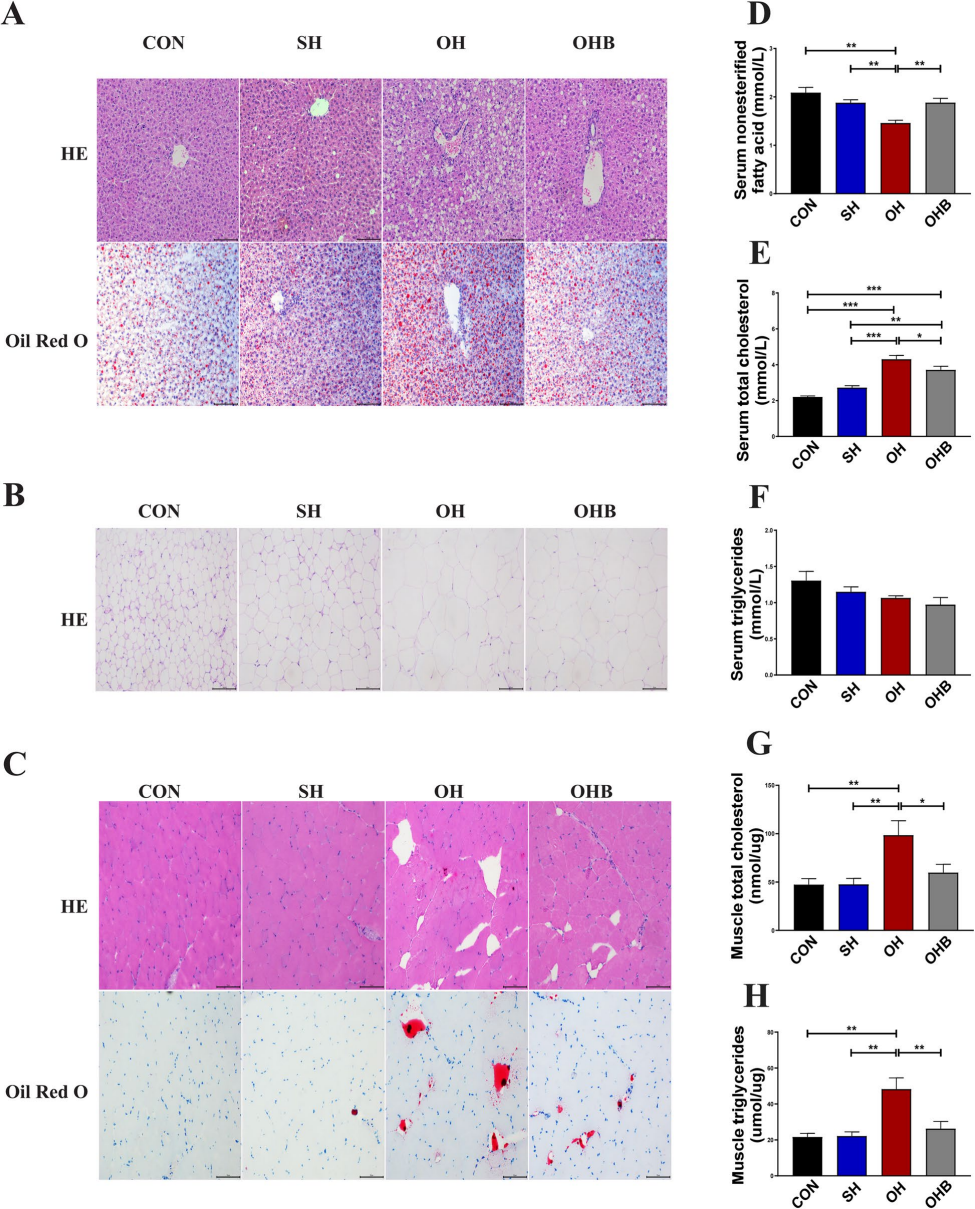
Lipid accumulation in serum and tissues in response to sodium butyrate. **A** Haematoxylin–eosin staining (HE) and Oil Red O staining in the liver (*n* = 3). **B** Haematoxylin–eosin staining in adipose tissue (*n* = 3). **C** Haematoxylin–eosin and Oil Red O staining in muscle (*n* = 3). **D-F** The concentrations of (**D**) nonesterified fatty acids, (**E**) total cholesterol and (**F**) triglycerides in the serum of the indicated mice (*n* = 10–12). **G-H** The concentrations of (**G**) total cholesterol and (**H**) triglycerides in the muscle tissue of the indicated mice (*n* = 6). CON: normal control diet, SH: sham operated + HFD, OH: OVX + HFD, OHB: OVX + HFD + NaB. Data are the means ± SEMs, and data were analysed by ANOVA (*p* < 0.05), *p < 0.05, **p < 0.01, ***p < 0.001

To understand how NaB improved energy expenditure and insulin sensitivity, we measured blood lipids and lipid deposition in liver, fat, and muscle tissues. Hepatic steatosis was evaluated based on HE and Oil Red O staining, where lipid deposition in hepatocytes was shown as white and red lipid droplets. The number and size of the lipid droplets measured using imaging software were significantly increased in all HFD-fed groups compared to the control, with the OH group showing the highest values among the three groups. The OHB group had a similar number and size of droplets to the SH group, but they were significantly lower in both groups than in the OH group (Fig. 3A, S2A-B). The size of fat cells was evaluated based on HE staining, which was shown as a fuchsia cell outline. The fat cell sizes measured using imaging software were significantly larger in all HFD-fed groups than in the control group, with the highest size in the OH group. The OHB group had cell sizes between those of the SH and OH groups (Fig. 3B, S2C). Lipid accumulation in muscle was consistent with hepatic HE and Oil Red O staining. The area of the lipid droplets was significantly increased in OVX mice compared to the control and SH groups and was reduced by NaB treatment (Fig. 3C, S2D-E). In addition, lipid parameters in serum and muscle tissue were measured for further evidence. The serum concentrations of nonesterified fatty acids (NEFA) and TC were significantly increased in the OH group, but NEFA and TC were significantly decreased in the OHB group (Fig. 3D, E). The concentrations of HDL-C and LDL-C in serum were higher in the OH group, but there was no significant difference with NaB treatment (data not shown). Serum TG levels in all groups were not shown to be altered by a high-fat diet or NaB treatment (Fig. 3F). The contents of TC and TG in OH mouse muscle were increased significantly, which could be reduced by NaB treatment (Fig. 3G, H).

### Sodium butyrate improves lipid metabolism and mitochondrial biogenesis

**Fig. 4:**
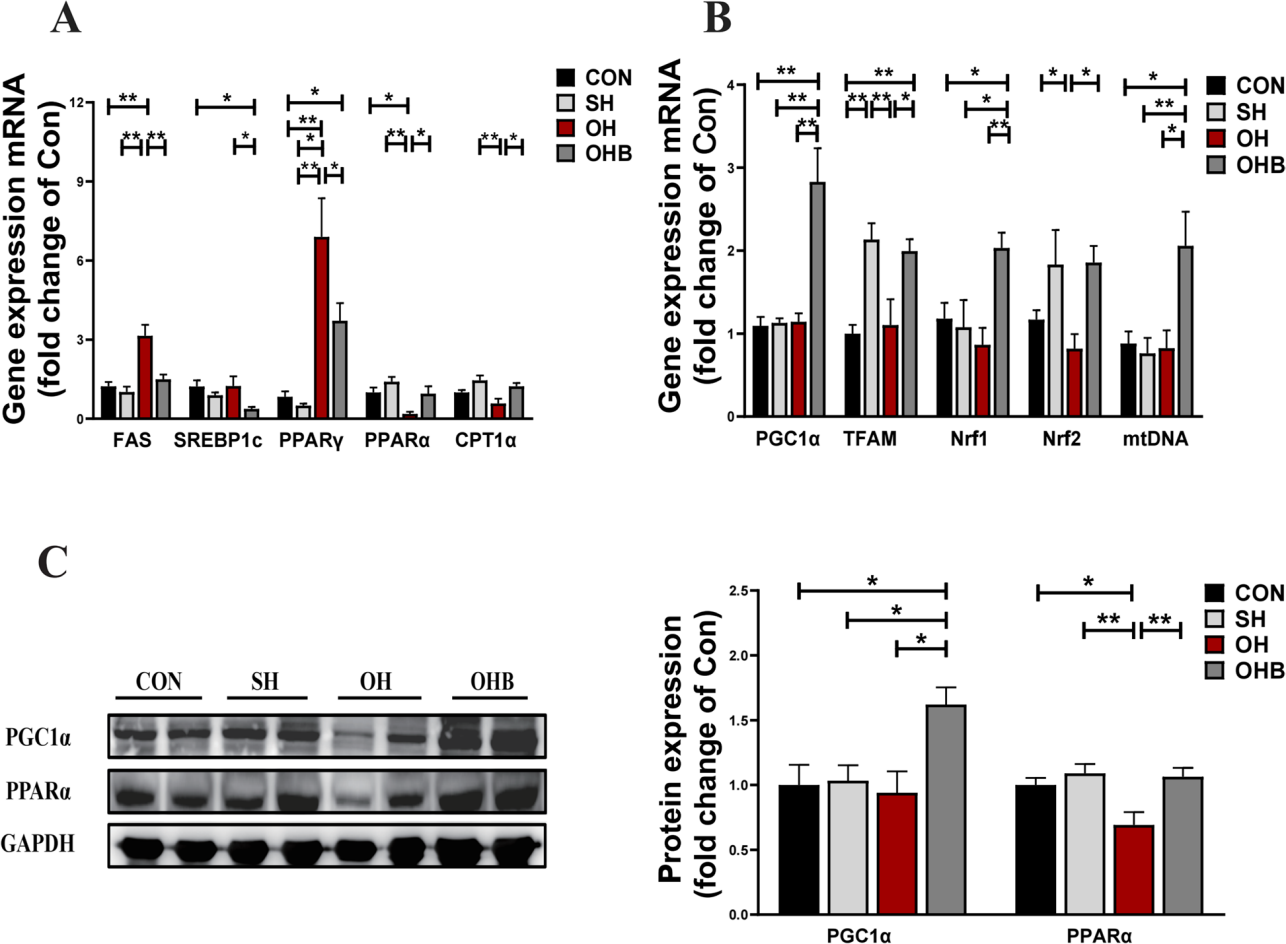
Lipid metabolism and mitochondrial function in NaB-treated mice. **A** The mRNA expression of key genes involved in lipid synthesis and lipolysis in skeletal muscle. **B** The mRNA expression of key genes involved in mitochondrial biogenesis in skeletal muscle. **C** The protein expression of PGC1α and PPARα in skeletal muscle (*n* = 6–8). Data are the means ± SEMs. The NaB effect was analysed by ANOVA (*p* < 0.05), *p < 0.05, **p < 0.01, ***p < 0.001

To understand the mechanisms responsible for muscle energy metabolism in response to NaB treatment, lipogenesis and fatty acid oxidation markers were examined in muscle tissue. The expression levels of lipogenesis markers, i.e., sterol regulatory element-binding transcription factor 1c (SREBP1c), fatty acid synthase (FAS), and the nuclear receptor and transcription factor peroxisome proliferator-activated receptor gamma (PPARγ), were decreased by NaB treatment, with OH mice showing the highest levels due to oestrogen deficiency compared to those of control and SH mice. In contrast, gene expression of the fatty acid oxidation marker genes peroxisome proliferator-activated receptor alpha (PPARα) and CPT1α showed the lowest levels in OH mice among the groups, but these levels were reversed by NaB treatment (Fig. 4A, C). Consistent with previous studies, oestrogen deficiency leads to impaired mitochondrial biogenesis [13], as assessed by the reduced peroxisome proliferator-activated receptor-γ co-activator 1α (PGC1α) levels. In this study, increased expression of transcription factor a mitochondria (TFAM), nuclear respiratory factor 1 (Nrf1), nuclear factor erythroid derived 2-like 2(Nrf2), mitochondrial DNA (mtDNA) and PGC1α, which are mitochondrial biogenesis markers, was found to be responsible for mitochondrial biogenesis in response to NaB treatment (Fig. 4B, C). These results indicated that NaB treatment improved lipid metabolism and mitochondrial biogenesis.

### Sodium butyrate improves mitochondrial respiratory function potentially through the ERα pathway

**Fig. 5:**
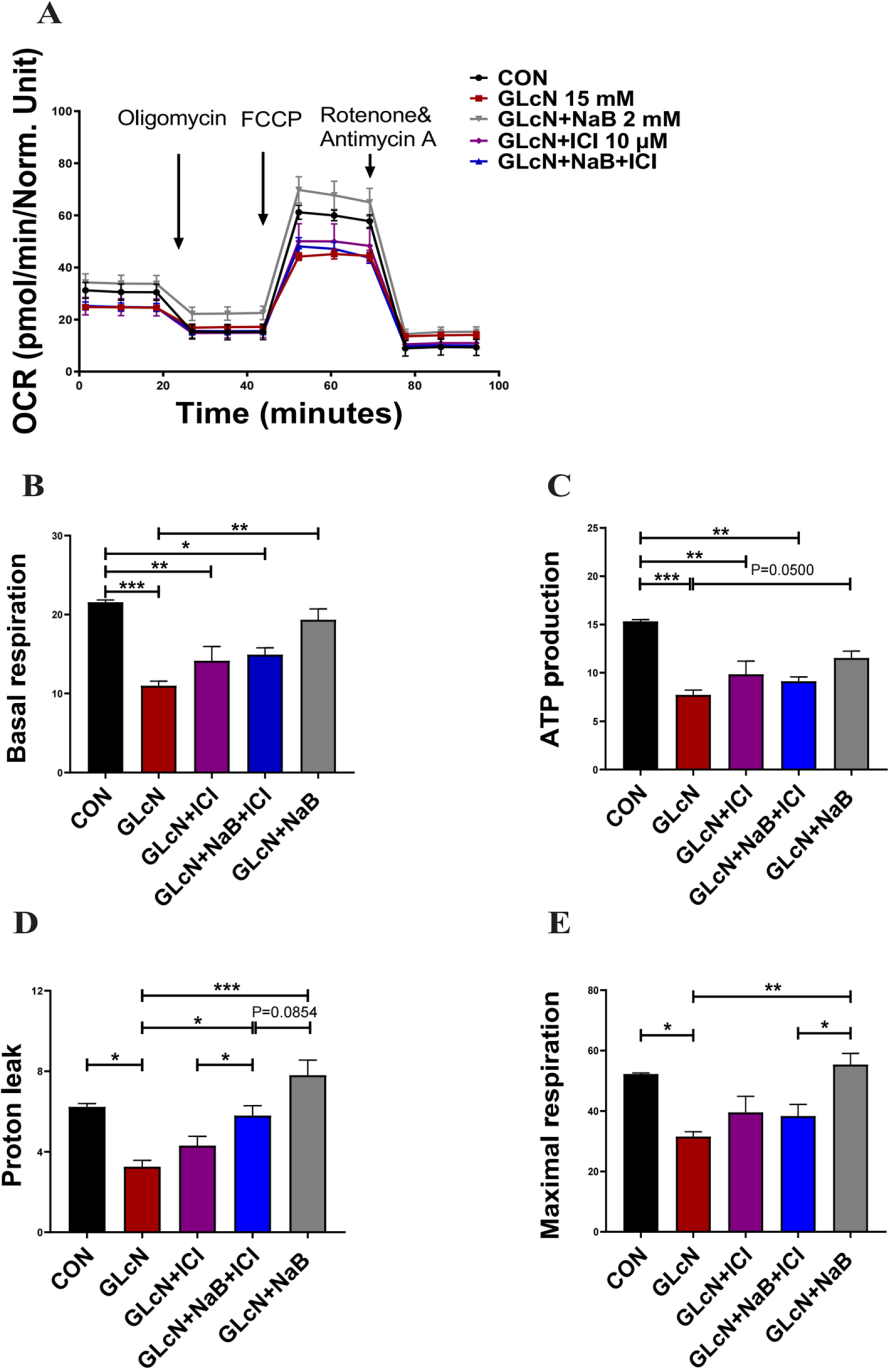
Mitochondrial respiratory function in NaB-treated C2C12 cells. **A** C2C12 cells treated with GLcN alone or in combination with NaB and/or fulvestrant (ICI) were consecutively injected with oligomycin, carbonyl cyanide p-(trifluoromethoxy) phenylhydrazone (FCCP), or rotenone/antimycin A, and the oxygen consumption rates (OCRs, pmol/min/mg protein) were recorded. **B-E**, (**B**) Cell basal respiration, (**C**) ATP production, (**D**) proton leak, and (**E**) maximal respiration. Mitochondrial respiration was calculated from the bioenergetic analysis. Data were normalized to protein concentration units per well prior to statistical analysis. Data are the means ± SEMs. The NaB effect was analysed by ANOVA (*p* < 0.05), *p < 0.05, **p < 0.01, ***p < 0.001

Next, the action of NaB in mitochondrial respiration was measured by Seahorse in C2C12 cells (Fig. 5A). Cellular basal respiration (i.e., mitochondrial complex I/III-linked respiration), ATP production, proton leak, and maximal respiration were all decreased with glucosamine treatment but were restored by NaB treatment (Fig. 5B-E). Furthermore, a glycolytic stress test by Seahorse was performed to measure the extracellular acidification ratio (ECAR). Basal glycolytic metabolism and glycolytic capacity in glucosamine-exposed cells were higher than those in control cells. NaB treatment recovered glycolytic homeostasis, indicating the critical role of NaB in glycolytic metabolism (Figure S3). Interestingly, compared to the glycolytic changes, the opposite trend of mitochondrial respiration indicated the potential role of NaB in metabolic reprogramming.

To investigate whether oestrogen receptor alpha participates in the effect on the mitochondrial bioenergetics of NaB, fulvestrant was used as a nonselective inhibitor of the oestrogen receptor (Fig. 5A). The results showed reduced maximal respiration and proton leakage in fulvestrant-treated C2C12 cells in contrast to NaB treatment (Fig. 5D-E). These data indicated that NaB could restore GLcN-induced mitochondrial dysfunction in C2C12 cells. In addition, oestrogen receptors are possibly involved in part of the regulation of NaB on mitochondrial oxidative phosphorylation.

### Sodium butyrate increases the expression of ERα and AMPK

**Fig. 6:**
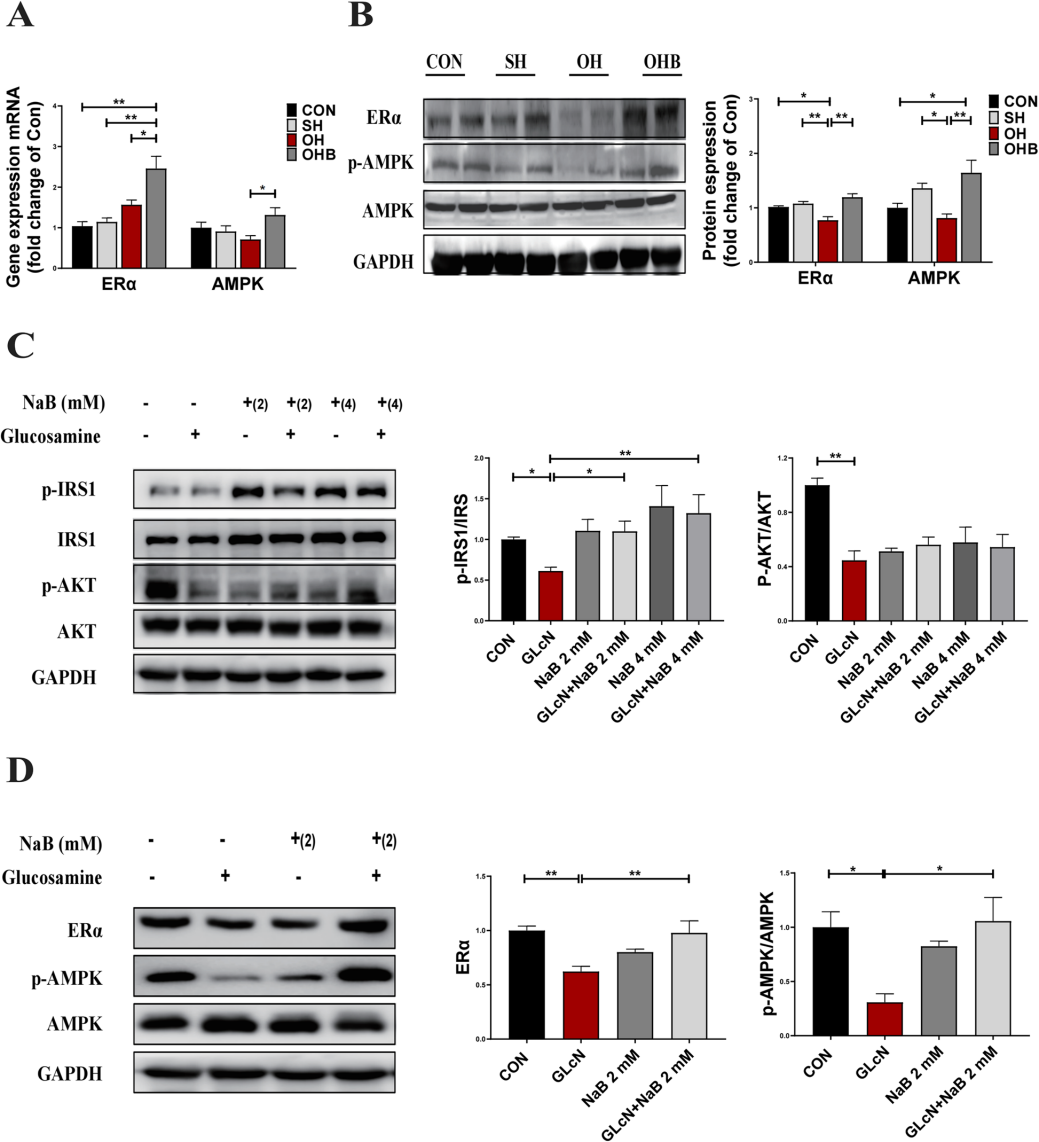
The expression of ERα and AMPK in NaB-treated muscle or C2C12 cells. **AB**,** B** The effect of NaB on ERα and AMPK in the skeletal muscle of mice. **A** The mRNA expression of ERα and AMPK (*n* = 8–10). **B** The protein expression of ERα and AMPK (*n* = 6). **C,D** The effect of NaB on the protein expression of key genes involved in the insulin signalling pathway and the ERα/AMPK signalling pathway induced by glucosamine exposure in C2C12 cells. **C** The relative protein expression of phosphorylated IRS1 and phosphorylated AKT. **D** The protein expression of ERα and the activation of AMPK in the presence or absence of NaB. Data are the means ± SEMs, and data were analysed by ANOVA (*p* < 0.05), *p < 0.05, **p < 0.01, ***p < 0.001

Then, ERα expression and AMPK activation were examined in skeletal muscle. The protein expression levels of ERα and the activation of AMPK were weaker in OH mice than in SH mice, with no difference in mRNA levels. However, the expression of ERα and the activation of AMPK at both the mRNA and protein levels were higher in the OHB mice than in the OH mice (Fig. 6A-B). Next, the in vitro results further verified the results already shown in vivo. The protein expression levels of activated insulin receptor substrate 1 (IRS1) and activated protein kinase B (AKT), markers of insulin resistance, were decreased in GLcN-treated cells, while they were increased by NaB treatment in a dose-dependent manner, although there were no statistically significant differences in AKT (Fig. 6C). The expression of ERα and the activation level of AMPK in vitro were observed to be consistent with the in vivo findings and were weaker in GLcN-treated cells than in control cells and higher in NaB-treated cells than in GLcN-treated cells (Fig. 6D). These results indicated that NaB could stimulate the expression of ERα and the activation of AMPK in muscle in vivo and in vitro.

### Sodium butyrate mediates the ERα/AMPK signalling pathway

**Fig. 7:**
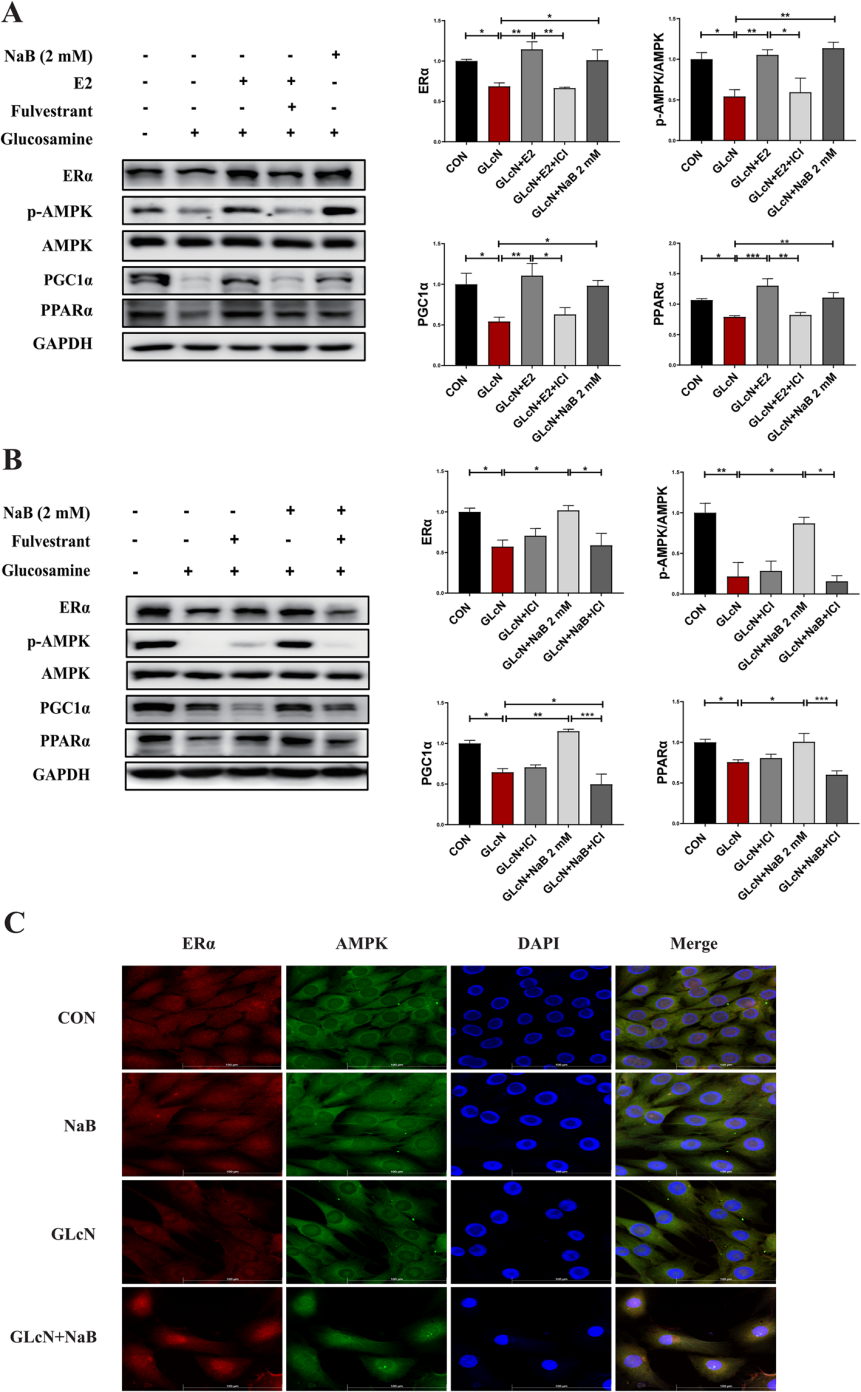
NaB activated AMPK via ERα in NaB-treated C2C12 cells. Effect of NaB on ERα and AMPK activation in the presence or absence of E2 or fulvestrant (ICI) induced by glucosamine exposure in C2C12 cells. **A** The protein expression of ERα and the activation of AMPK, PGC1α and PPARα in the presence or absence of E2, fulvestrant and NaB. **B** The protein expression of ERα and the activation of AMPK, PGC1α and PPARα in the presence or absence of NaB and fulvestrant. **C** Confocal microscopy analysis was detected by the corresponding fluorescence secondary antibodies in C2C12 cells treated with GLcN or NaB. ERα (in red), AMPK (in green), and nuclei were stained with DAPI (in blue). The merged images denote the colocalization of ERα and AMPK (in yellow). Data are the means ± SEMs. The NaB effect was analysed by ANOVA (*p* < 0.05), *p < 0.05, **p < 0.01, ***p < 0.001

To determine whether NaB activates the ERα-AMPK signalling pathway, gene expression changes were first examined under ERα function activation by E2 in GLcN-treated C2C12 cells (Fig. 7A). Increased expression levels of ERα and AMPK activation were found in E2-treated cells. The expression of PGC1α and PPARα, which lie downstream of AMPK, also showed significantly increased expression. When ERα function was inhibited by fulvestrant, a contrary result was found regarding the decreased expression of those genes. Consistent with the effect resulting from E2, increased expression of those genes was found in NaB-treated cells. Furthermore, gene expression was examined when fulvestrant was added to NaB-treated cells (Fig. 7B). The effect of NaB on increasing AMPK activation and downstream gene expression was weakened due to functional ERα depletion. Moreover, confocal microscopy analysis was performed to indicate the location and interaction between ERα and AMPK in C2C12 cells (Fig. 7C, S4A-B). The results showed that ERα (stained with red fluorescence) was evenly distributed in the cytoplasm and nucleus in control cells. The fluorescence intensity was markedly weaker in GLcN-treated cells than in control cells, while it was higher in NaB-treated cells than in GLcN-treated cells. Interestingly, with NaB treatment, ERα was more prone to localize in the nucleus, contrary to with GLcN treatment. AMPK (stained with green fluorescence) was mainly located in the cytoplasm without evidence of translocation in all cells. However, the fluorescence intensity was increased by NaB treatment in GLcN-treated cells. As expected, ERα and AMPK were colocalized in the cytoplasm, as shown by the merged images in yellow.

These results supported the hypothesis that NaB stimulated AMPK activation through ERα, thereby inducing PGC1α and PPARα gene expression and activation, increasing mitochondrial biogenesis and oxidative phosphorylation, enhancing mitochondrial aerobic respiratory activity and weakening glycolysis capacity, resulting in the amelioration of metabolic disorders.

## Discussion

Menopause is a signature event in a woman’s life, characterized by age-related hormonal and metabolic environmental changes that occur at an average age of 51 years [26]. Oestrogen deficiency and relative androgen excess are attributed a central role in the onset of postmenopausal MetS [27, 28]. In this study, we also found that oestradiol levels, the prevalence rate of MetS and risk factors of MetS changed after 50 years of age. Importantly, there is a correlation between metabolic syndrome and oestradiol levels in females. All of the above findings support the existence of sexual dimorphism and suggest that oestrogen might play a critical role in maintaining metabolic homeostasis in females.

Our previous studies have found that butyrate contributes to non-alcoholic fatty liver disease in premenopause due to oestrogen deficiency with lower butyrate contents in patients and mice [29]. It was also reported that NaB improved insulin sensitivity in diet-induced mice [7, 9]. However, the mechanism by which NaB improves insulin sensitivity remains unclear, especially in oestrogen-deficient animals or humans. In current study, mice were oophorectomized to simulate menopause and oestrogen deficiency, as previously reported [30, 31]. We observed increased weight, elevated lipid levels, and decreased insulin sensitivity induced by oestrogen deficiency. NaB treatment could ameliorate abdominal obesity and insulin resistance in diet-induced oestrogen-deficient mice.

Previous studies have reported that butyrate alleviates obesity and insulin resistance by increasing energy expenditure in high-fat diet-fed (HFD) mice [6–9]. Oestrogen deficiency reduces energy expenditure in postmenopausal women and ovariectomized mice [3, 32, 33]. We found that NaB treatment enhanced energy expenditure without increasing calorie intake and activity in diet-induced oestrogen-deficient mice. Notably, less activity accompanied by low energy expenditure was observed in oestrogen-deficient mice. The lower activity of oestrogen-deficient mice may be due to the increased lipid accumulation in muscle in response to hormone changes [34]. NaB treatment enhanced energy expenditure without increasing activity, which is consistent with reports that energy expenditure reduction is not due only to decreased activity in OVX mice [3, 35].

In addition, we confirmed that NaB treatment increased lipid metabolism and mitochondrial biogenesis in skeletal muscle in diet-induced oestrogen-deficient mice. Skeletal muscle plays a significant role in whole-body energy balance, and muscle disorders have been reported to be closely associated with obesity, insulin resistance, and type 2 diabetes [10]. NaB treatment decreased lipid accumulation and the concentrations of TG and TC in skeletal muscle in HFD-fed mice as previously reported [36], but TC concentrations decreased and NEFA concentrations increased in serum by NaB treatment in diet-induced oestrogen-deficient mice, which was not consistent with the lack of change in HFD-fed mice [36]. This finding might be explained by the substrate utilization shift of muscle [37]. Furthermore, NaB treatment downregulated lipid synthesis genes, upregulated lipolysis marker genes, and increased mitochondrial biogenesis in skeletal muscle in diet-induced oestrogen-deficient mice, which were reported in muscle or liver in HFD-fed mice [7, 36, 38]. Mitochondrial biogenesis involves both proliferation and differentiation, which mean that mitochondrial content increases and mitochondrial capacities improve, respectively. Previous researchers have reported sexual dimorphism of mitochondrial functionality in skeletal muscle, with greater mitochondrial differentiation and content in female rats than in males. Therefore, females can better adapt to metabolic energy situations, mainly through alterations involving greater oxidative phosphorylation capability [13]. PGC1α can activate TFAM through the induction of Nrf1 and Nrf2 and can then drive the transcription and replication of mitochondrial DNA. NrF1 activation triggers the expression of proteins involved in the regulation of mitochondrial respiratory chain subunits, which cooperate with PGC1α and TFAM to regulate the function of mitochondrial oxidation [13]. Our findings suggest that NaB treatment improved lipid metabolism and mitochondrial biogenesis, which are still critical reasons for the increased energy expenditure in diet-induced oestrogen-deficient mice.

Furthermore, we confirmed that NaB-ERα-AMPK pathway activation was critical for regulating metabolic homeostasis with oestrogen deficiency. We observed significant AMPK activation and PGC1α expression upregulation in both muscle and C2C12 cells. In agreement with previous publications, the action of butyrate was attributed to AMPK activation [9]. AMPK is well known as a master metabolic switch, and its activity correlates well with improving obesity and insulin sensitivity by regulating mitochondrial function and fatty acid metabolism in skeletal muscle [39]. AMPK and downstream PGC1α have been confirmed to mediate the effects of NaB by increasing fatty acid oxidation and enhancing mitochondrial function [36, 38]. AMPK-PGC1α pathway activation contributes to the stimulation of mitochondrial biogenesis in insulin-resistant muscle [11]. In addition, the actions of E2 on activating AMPK and stimulating PPARα expression while downregulating genes that control lipogenesis (FAS and SREBP-1c) [39] were entirely consistent with our findings of decreased lipid accumulation in muscle from ovariectomized mice with NaB treatment. However, the mechanism by which NaB activates AMPK remains unknown. A recent study showed that oestrogen-induced AMPK activation was ER mediated [19]. ERs exist in two main forms (α and β), are expressed in a variety of tissues, are particularly highly expressed in insulin-sensitive tissue, and exhibit tissue specificity in expression and function. The expression and functionality of ERα were considered to be key determinants of oestradiol efficacy on metabolic health [17] in terms of the link between its impairment and the increased prevalence of specific features of MetS in male or female human subjects and whole body or muscle-specific knockout rodent models [17, 39, 40]. Oestrogen receptor ablation-induced loss of oestrogen action was a likely origin for reduced AMPK activation in skeletal muscle from ERα-KO mice [1], while in vivo results from the specific activation of ERα by PPT showed increased AMPK activation in muscle [41]. Thus, we proposed that NaB treatment could activate the ERα-AMPK signalling pathway in muscle to respond to metabolic disorders with oestrogen deficiency. Supporting this hypothesis was the fact that the level of AMPK activation increased subsequently to increase ERα expression by oestrogen action, while genes that control mitochondrial biogenesis and fatty acid metabolism, including PGC1α and PPARα, were also upregulated. Moreover, the same effect together with downstream genes was found in NaB-treated cells. In contrast, the reduction in the level of AMPK activation and the downregulation of gene expression were due to the expression or functional ablation of oestrogen receptor alpha. In addition, the protective effect of increasing mitochondrial oxidative capability and decreasing glycolysis in vitro by NaB treatment was reversed with treatment with an ERα receptor inhibitor. Moreover, ERα and AMPK were colocalized in the cytoplasm, and their expression increased in NaB-treated cells. However, more evidence is needed to verify the direct relationship between ERα and AMPK, and further research on the mechanism of NaB action on ERα regulation is needed. In general, all of the above results showed a link between NaB and ERα in muscle. Metabolism stimulated by AMPK activation might be directly mediated by ERα.

In conclusion, dietary butyrate supplementation can improve metabolic disorders caused by oestrogen deficiency, including obesity and insulin resistance. The action of butyrate is related to the promotion of energy expenditure through the improvement in oxidative phosphorylation of mitochondria in muscle cells. In addition, butyrate activates the NaB-ERα-AMPK signalling pathway in muscle and plays a role in relieving oestrogen deficiency-induced metabolic disorders. The fact that NaB is effective against oestrogen-deficient disorders may indicate its potential for the prevention and treatment of MetS in postmenopausal females.

## Supplementary Information

**Additional file 1: Figure S1.** Clinical data analyses of metabolic syndrome.Sex and age differences in prevalence of metabolic syndrome.Body Mass Index.The concentrations of serumhigh density lipid cholesterollow density lipoprotein cholesteroltotal cholesteroltriglyceridesblood glucoseglycated haemoglobin A1c in females and males in age groups.The concentrations of serum oestradiol in age groups.The concentrations of serum oestradiol in females with MetS or not. MetS: Metabolic syndrome, F: Female, M: Male. Data are the means ± SEMs, and data were analysed by ANOVA, unpaired t test, *p<0.05, **p<0.01, ***p<0.001.

**Additional file 2: Figure S2.** Lipid accumulation of tissues in NaB-treated mice.The area of white lipid droplets andred lipid droplets were counted in liver. The area proportion was normalized to normal control.Mean size of adipose cell.The area of white lipid droplets andred lipid droplets were counted in muscle. The area proportion was normalized to normal control.. CON: normal control diet, SH: sham operated+HFD, OH: OVX+HFD, OHB: OVX+HFD+NaB. Data are the means ± SEMs, and data were analysed by ANOVA, *p<0.05, **p<0.01, ***p<0.001.

**Additional file 3: Figure S3.** The time course for measurement of ECAR in C2C12 cells treated with or without glucosamine and/or NaB. The ECAR was measured under baseline conditions and after glucose, oligomycin and 2-Deoxy-D-glucoseinjection, as indicated by the arrowheads.Glycolysis.Glycolytic capacity. ECAR as calculated from the bioenergetic analysis. Data were normalized to protein concentration units per well prior to statistical analysis. Data are the means ± SEMs. The NaB effect was analysed by ANOVA, *p<0.05, **p<0.01, ***p<0.001.

**Additional file 4: Figure S4.** Confocal microscopy analyses in C2C12 cells with GLcN or NaB treatment.Mean fluorescence intensity of ERα.Mean fluorescence intensity of AMPK. Data are the means ± SEMs. The NaB effect was analysed by ANOVA, *p<0.05, **p<0.01, ***p<0.001.

**Additional file 5: Table S1.** qRT-PCR primer sequences

## Data Availability

The datasets supporting the conclusions of this article are included within the article and its additional files.

## Abbreviations

MetS: Metabolic syndrome
NaB: Sodium butyrate
SCFA: Short chain fatty acids
OVX: Ovariectomy
HFD: High-fat diet
CON: Normal control diet group;
SH: Sham operated + HFD group
OH: OVX + HFD group
OHB: OVX + HFD + NaB group
AMPK: AMP-activated protein kinase
ERα: Oestrogen receptor alpha
EREs: Oestrogen response elements
BMI: Body mass index
NEFA: Nonesterified fatty acid
TG: Triglyceride
TC: Total cholesterol
RER: Respiratory exchange ratio
EE: Energy expenditure
