# Supplementary figures and images for: Butyrate mitigates metabolic dysfunctions via the ERα-AMPK pathway in muscle in OVX mice with diet-induced obesity
Source: Cell Commun Signal. 2023 May 4;21:95. doi: 10.1186/s12964-023-01119-y (PMC10158218; doi:10.1186/s12964-023-01119-y)

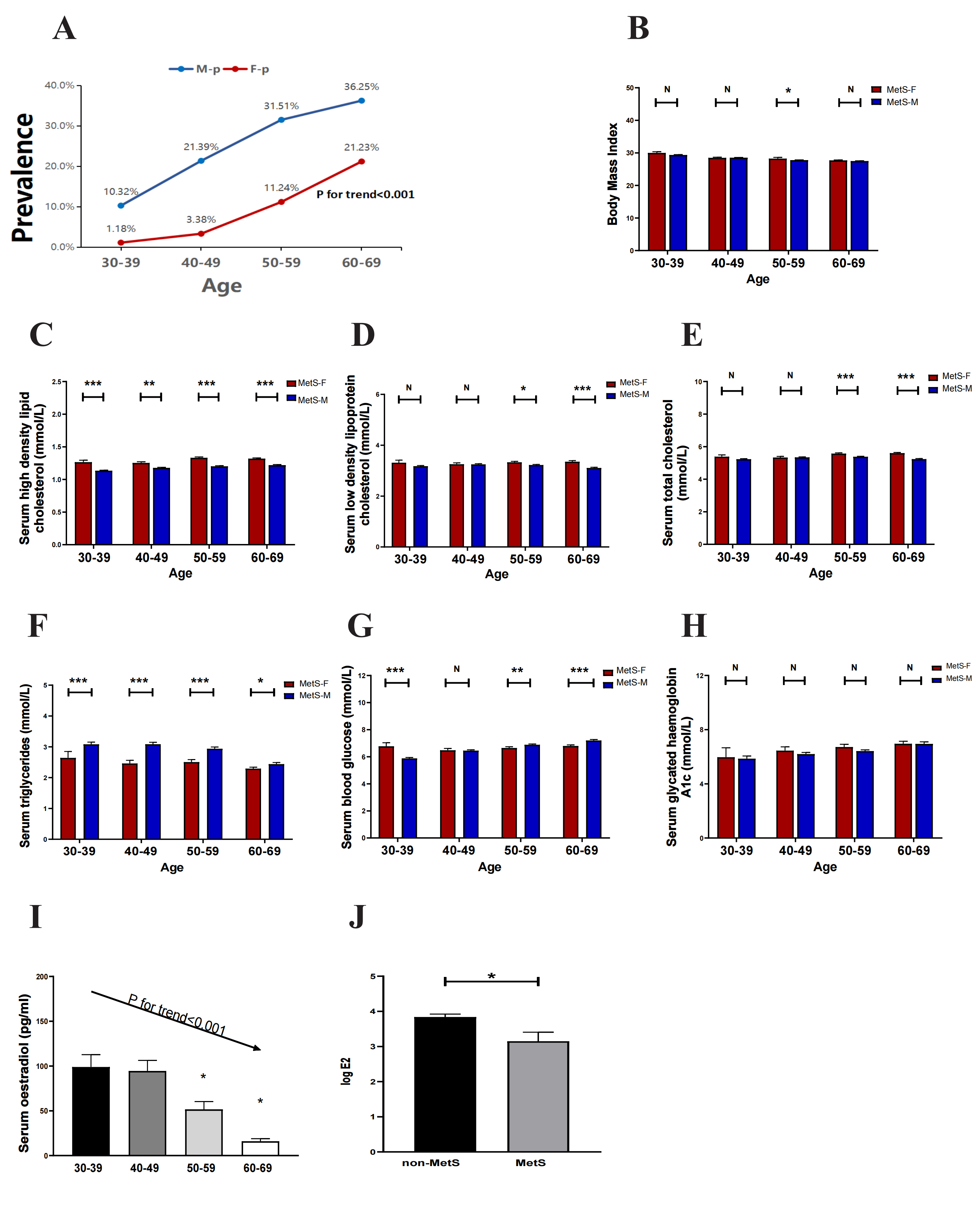

Supplement: Supplementary file 2 — Additional file 1: Figure S1. Clinical data analyses of metabolic syndrome.Sex and age differences in prevalence of metabolic syndrome.Body Mass Index.The concentrations of serumhigh density lipid cholesterollow density lipoprotein cholesteroltotal cholesteroltriglyceridesblood glucoseglycated haemoglobin A1c in females and males in age groups.The concentrations of serum oestradiol in age groups.The concentrations of serum oestradiol in females with MetS or not. MetS: Metabolic syndrome, F: Female, M: Male. Data are the means ± SEMs, and data were analysed by ANOVA, unpaired t test, *p<0.05, **p<0.01, ***p<0.001. [file 12964_2023_1119_MOESM1_ESM.tif]

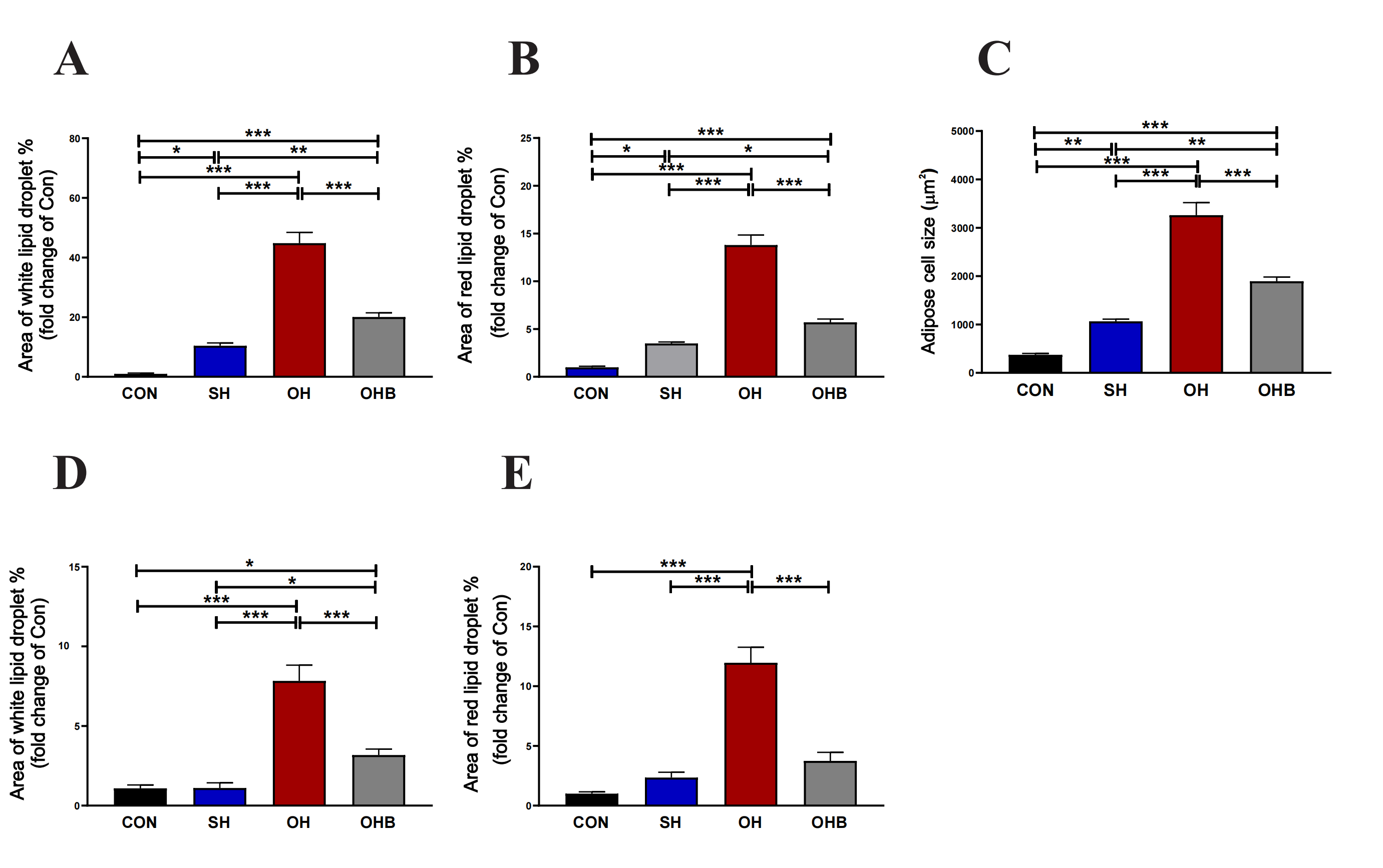

Supplement: Supplementary file 3 — Additional file 2: Figure S2. Lipid accumulation of tissues in NaB-treated mice.The area of white lipid droplets andred lipid droplets were counted in liver. The area proportion was normalized to normal control.Mean size of adipose cell.The area of white lipid droplets andred lipid droplets were counted in muscle. The area proportion was normalized to normal control.. CON: normal control diet, SH: sham operated+HFD, OH: OVX+HFD, OHB: OVX+HFD+NaB. Data are the means ± SEMs, and data were analysed by ANOVA, *p<0.05, **p<0.01, ***p<0.001. [file 12964_2023_1119_MOESM2_ESM.tif]

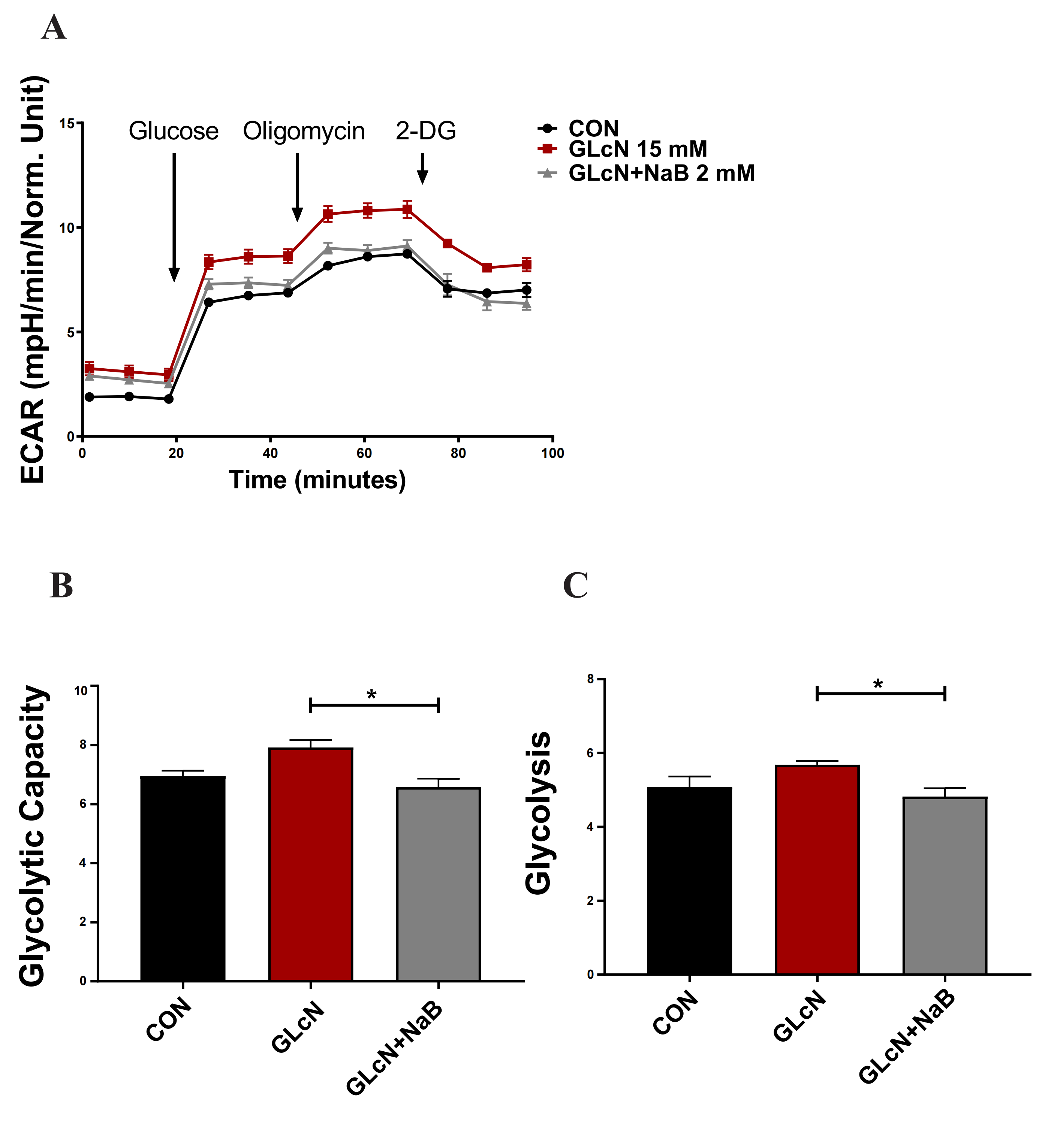

Supplement: Supplementary file 4 — Additional file 3: Figure S3. The time course for measurement of ECAR in C2C12 cells treated with or without glucosamine and/or NaB. The ECAR was measured under baseline conditions and after glucose, oligomycin and 2-Deoxy-D-glucoseinjection, as indicated by the arrowheads.Glycolysis.Glycolytic capacity. ECAR as calculated from the bioenergetic analysis. Data were normalized to protein concentration units per well prior to statistical analysis. Data are the means ± SEMs. The NaB effect was analysed by ANOVA, *p<0.05, **p<0.01, ***p<0.001. [file 12964_2023_1119_MOESM3_ESM.tif]

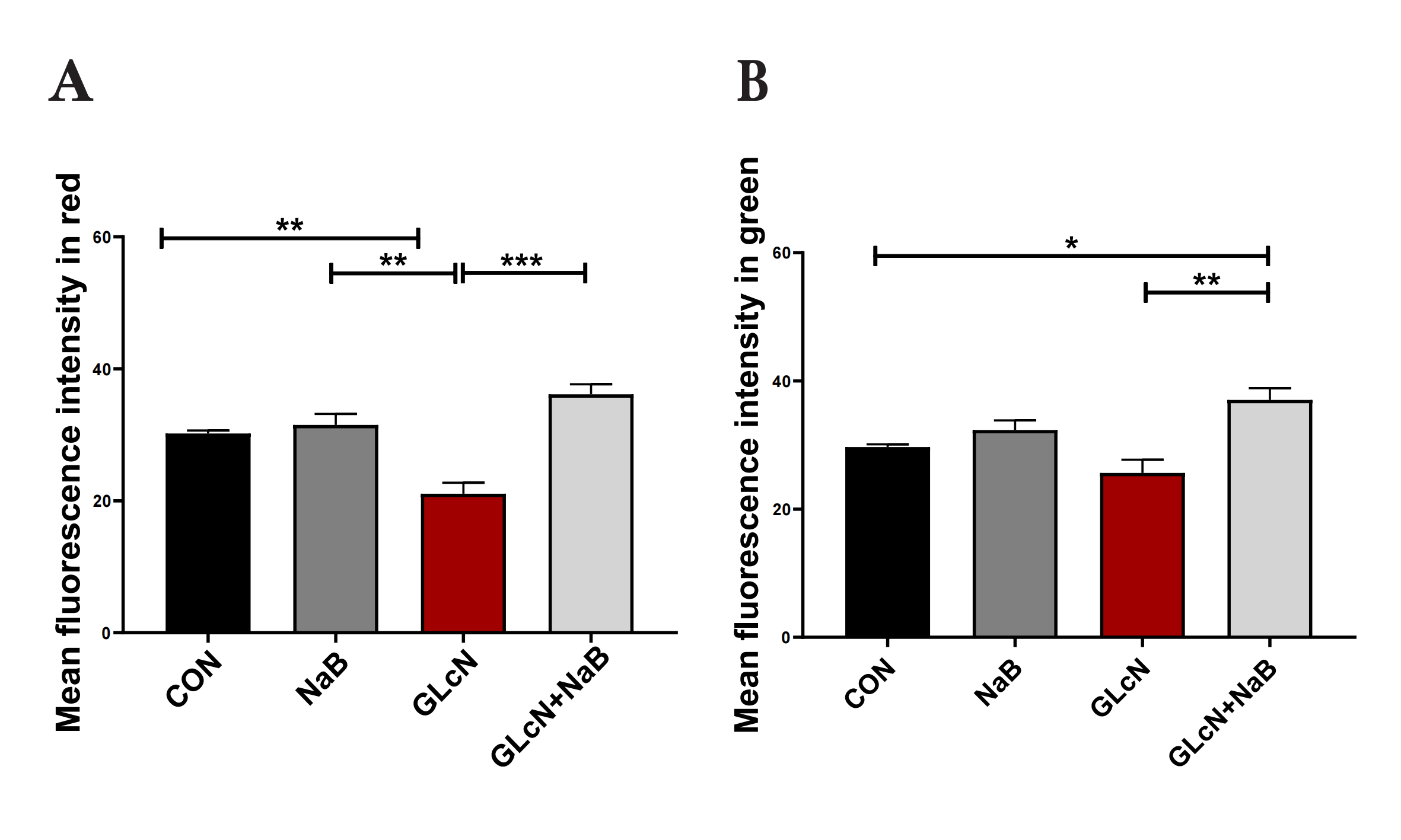

Supplement: Supplementary file 5 — Additional file 4: Figure S4. Confocal microscopy analyses in C2C12 cells with GLcN or NaB treatment.Mean fluorescence intensity of ERα.Mean fluorescence intensity of AMPK. Data are the means ± SEMs. The NaB effect was analysed by ANOVA, *p<0.05, **p<0.01, ***p<0.001. [file 12964_2023_1119_MOESM4_ESM.tif]
