# Supplementary material for: Butyrate mitigates metabolic dysfunctions via the ERα-AMPK pathway in muscle in OVX mice with diet-induced obesity
Source: Cell Commun Signal. 2023 May 4;21:95. doi: 10.1186/s12964-023-01119-y (PMC10158218; doi:10.1186/s12964-023-01119-y)
Supplement: Supplementary file 6 — Additional file 5: Table S1. qRT-PCR primer sequences [file 12964_2023_1119_MOESM5_ESM.docx]

**Table 1 qRT-PCR primer sequences**

| **Gene** | **Forward Primer Sequence (5' to 3')** | **Reverse Primer Sequence (5' to 3')** |
| --- | --- | --- |
| PPARα | AGAGCCCCATCTGTCCTCTC | ACTGGTAGTCTGCAAAACCAAA |
| PPARγ | TCGCTGATGCACTGCCTATG | GAGAGGTCCACAGAGCTGATT |
| SREBP1c | TGACCCGGCTATTCCGTGA | CTGGGCTGAGCAATACAGTTC |
| CPT1α | CTCCGCCTGAGCCATGAAG | CACCAGTGATGATGCCATTCT |
| FAS | GCGGGTTCGTGAAACTGATAA | GCAAAATGGGCCTCCTTGATA |
| AMPK | GTCAAAGCCGACCCAATGATA | CGTACACGCAAATAATAGGGGTT |
| PGC1α | TATGGAGTGACATAGAGTGTGCT | CCACTTCAATCCACCCAGAAAG |
| TFAM | ATTCCGAAGTGTTTTTCCAGCA | TCTGAAAGTTTTGCATCTGGGT |
| Nrf1 | AGCACGGAGTGACCCAAAC | TGTACGTGGCTACATGGACCT |
| Nrf2 | TCTTGGAGTAAGTCGAGAAGTGT | GTTGAAACTGAGCGAAAAAGGC |
| Cox1 | ACTATACTACTACTAACAGACCG | GGTTCTTTTTTTCCGGAGTA |
| Cyclophilin A | ACACGCCATAATGGCACTGG | CAGTCTTGGCAGTGCAGAT |
| ERα | CCCGCCTTCTACAGGTCTAAT | CTTTCTCGTTACTGCTGGACAG |
| GAPDH | TGACCTCAACTACATGGTCTACA | CTTCCCATTCTCGGCCTTG |
